# Incidence of remote consultation on general practitioners’ antibiotic prescriptions in 2021: a French observational study

**DOI:** 10.3399/BJGPO.2023.0196

**Published:** 2024-05-15

**Authors:** Cécile Rullier, Vincent Tarazona, David De Bandt

**Affiliations:** 1 Family medicine department, University of Paris Saclay, Versailles-Saint-Quentin-en-Yvelines, Paris, France

**Keywords:** family medicine, prescribing, infectious illness, remote consultation, antibacterial agents, general practice

## Abstract

**Background:**

In patients with infectious diseases, remote consultation (RC) may be questionable compared with face-to-face office consultation (OC), not only because of the lack of physical examination but also because of the risk of overprescribing antibiotics (ATBs).

**Aim:**

To analyse ATB prescription in OC versus RC in a sample of French GPs.

**Design & setting:**

This is a retrospective observational cohort study in general practice in 2021. Anonymised data were collected from voluntary GPs.

**Method:**

The influence of the mode of consultation on ATB prescription was analysed using a χ² test. A secondary multivariate analysis investigated the factors influencing the use of OC or RC in patients who received at least one ATB.

**Results:**

In total, 35 503 consultations with an identifiable rating were included, corresponding to seven doctors' activities, practising with five locums and three residents. ATBs were prescribed in 10.41% of RCs and 6.77% of OCs (*P*<0.01). RC was associated with more frequent prescription of ATBs for respiratory and ear, nose, and throat (ENT) viral infections and urinary tract infections. For patients aged 20–40 years, ATB prescription was more associated with RC.

**Conclusion:**

RC is associated with a more frequent ATB prescription than OC, mostly for patients aged 20–40 years, who are most likely to use new technologies; and for urinary tract infections or respiratory and ENT viral infections. Further studies on RC outcomes should be conducted to better analyse the impact of RC on the prescribing of ATBs.

## How this fits in

Remote consultation (RC) has become a common practice for French GPs since March 2020 in response to the confinement measures for the COVID-19 pandemic. There is a suspected risk of overprescribing antibiotics (ATBs) in RCs because of the lack of physical examinations, but there is still insufficient solid evidence. This study analysed the frequency of ATB prescription in RCs versus face-to-face office consultations (OC) in the activity of GPs in 2021. ATBs were more frequently prescribed in RCs than in OCs. General practitioners should consider limiting the prescription of antibiotics during teleconsultations before this issue is taken up by public health policymakers.

## Introduction

Remote consultation (RC) is defined by article R6316-1 of the Public Health Code in France, and allows any doctor *'to give a remote consultation to a patient*' by video transmission.^1^ The RC is reimbursed in France in the same way as face-to-face office consultation (OC) since 15 September 2018. The RC must respect the patient’s care pathway, allow the possibility of seeing the patient face to face if necessary, and be carried out under conditions allowing for confidential exchanges with a follow-up in the medical record. Certain exceptions exist, particularly in the context of emergencies.

RC has developed significantly in France as of March 2020 because of the COVID-19 pandemic. It has benefited from more flexible rules such as full coverage by the French social security system, a derogation from the patient’s care pathway, a derogation from the video transmission obligation until June 2021, and the authorisation to exceed the limit on the annual number of teleconsultations. RCs accounted for almost one-quarter of GP consultations during lockdown and dropped to stabilise at 4% in 2021. Three out of four physicians have implemented RCs since the start of the COVID-19 epidemic, while less than 5% practised it before.^2^

Presented by the software developer as a time-saver for physicians, and facilitating access to care for the patients, the RC appears as an innovative tool to address the problems of medical deserts. However, the quality of the care provided via this type of consultation is often questioned. The limitations include the lack of physical examination and the dehumanisation of the relationship.^3^

In the case of infectious pathologies that may lead to an antibiotic prescription (ATB), it seems reasonable to consider the physical examination as necessary.^4^ The French Academy of Medicine warns against the risk of overprescribing ATBs in RCs.^5^ The consumption of ATBs in France in 2019 was 30% higher than the European average despite a downward trend since 2009. The year 2021 was marked by an increase in ATB consumption.^6,7^ GPs are the main prescribers.

The impact of RC on the prescription of ATBs in primary care seems to vary greatly from one country to another with little data available.^8^ This observation has leds us to question the use of ATBs via RC in the French national context where the prescription of ATBs is high, and the use of RC recent. What is the impact of RC on the prescription of ATBs in general practice? The main objective of this study was to compare ATB prescription in RC with OC in the activity of GPs in primary care. The second objective was to identify the factors associated with the mode of consultation of patients who received a prescription of ATBs by their GP.

## Method

### Study design

This is a retrospective observational study of medical practices, conducted using anonymised patient data from the Weda professional medical software of GPs in 2021. Statistical analyses were performed with R Studio (23.08.0).

### Population

For the sample size calculation, we used available data showing that 40% of French people received at least one ATB in 2016, and that RC represented around 4% of all consultations in 2021.^2,9^ Based on the null hypothesis that there is no difference in the prescription of ATBs in OC versus RC, it was necessary to include approximately 200 consultations with prescription of an ATB in RC and approximately 2000 consultations with prescription of an ATB in OC.

Only physicians using the Weda professional software allowing data extraction were able to participate in this study. It is certified for data protection and is one of the most used software programmes in France.^10^ Since RC is a recent practice in France, the published data were not sufficient to calculate the number of participating physicians to recruit. It was therefore decided to recruit practitioners by solicitation on the Weda networks and then by snowball effect. The objective was to obtain varied physician profiles.

In order to avoid a possible season effect, the consultation data were extracted for the entire year of 2021. Consultations for which the type of consultation was not identifiable, without valid billing code, or where the patients had voluntarily terminated their data sharing with their practitioner, were excluded. The decision to stop recruiting practitioners was made when the number of consultations needed to be included had been obtained. The consultations by locums and residents were pooled with the consultations of the installed practitioner.

### Data management and statistical analysis

For each GP included, all consultations conducted during 2021 were sorted by billing code type to determine the total number of face-to-face consultations coded as OC and the total number of remote consultations coded as RC.^11,12^ All consultations involving an ATB prescription were extracted, screened, and anonymised by the principal investigator under the supervision of the GPs. The mode of consultation (either OC or RC), age (categorised by 20-year age groups), sex, presence of a long-term condition (LTC), existence of an allergy to β-lactams, and consultation result leading to ATB prescription were documented. Consultation results were derived from raw data and were coded according to the consultation results scheme of the French Society of General Medicine.^13^ When it was unknown or unclear after debriefing with the GP, it was classified as 'other'. The pathologies were then grouped by categories according to ATB prescription recommendation.^14^

The main evaluation criterion of our study was the prescription of ATB according to the mode of consultation in RC or OC and the statistical analysis was carried out by Pearson’s χ^2^ test.

Secondary analyses were carried out for patients who received an ATB prescription during the year to look for correlations between the mode of consultation and the characteristics of the consultations. A multivariate analysis with a logistic regression model was used with adjustment for age, sex, LTC, consultation results, and β-lactam allergy. The reference age category for comparisons was 20–40 years because of their proximity to informatics tools. The reference consultation result was otitis media because of the importance of the clinical examination to make the diagnosis. Other adjusted variables were binary.

## Results

### Study population

**Figure 1.**
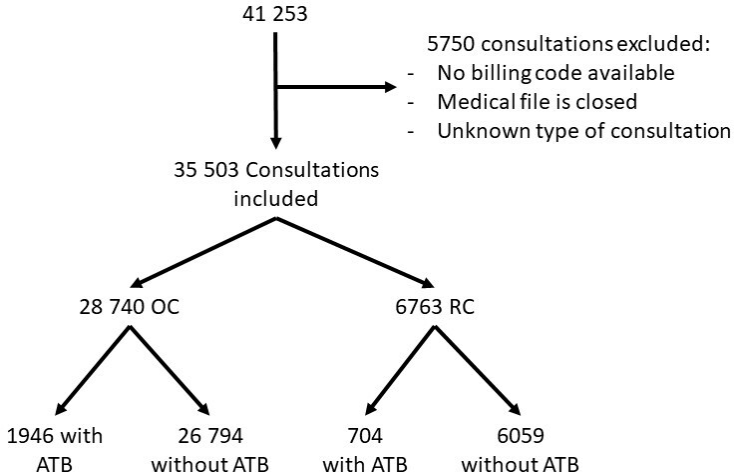
Flow chart for GPs' consultations. ATB = antibiotic prescription. OC = office consultation. RC = remote consultation

A total of 41 253 consultations were recorded in 2021 but 5750 consultations were excluded owing to the lack of an identifiable billing code (Figure 1). Billing codes used for all consultations screened in 2021 are available in Supplementary Table S1. In all, 35 503 consultations were included, which were divided into 28 740 OC (80.95%) and 6763 RC (19.05%). Data were collected from seven voluntary GPs, practising with five locums and three residents, who were located in two distinct consultation sites; three were in Ile de France and four in the Rhône region. No practitioner has been excluded (Table 1). Four patients’ data were excluded because data sharing of their medical file has been terminated.

**Table 1.** GPs' characteristics

GP	Sex	Age	Mode of Practice	Activity area	Have resident	Région	Centre
1	M	34	Pluriprofessional office	Urban	Yes	Ile de France	1
2	F	36	Pluriprofessional office	Urban	No	Ile de France	1
3	M	63	Alone office	Urban	No	Ile de France	2
4	F	32	Pluriprofessional office	Urban	No	Rhône	3
5	M	62	Pluriprofessional office	Urban	Yes	Rhône	4
6	M	44	Pluriprofessional office	Urban	No	Rhône	5
7	M	36	Pluriprofessional office	Urban	Yes	Rhône	6

### Data analysis

**Figure 2.**
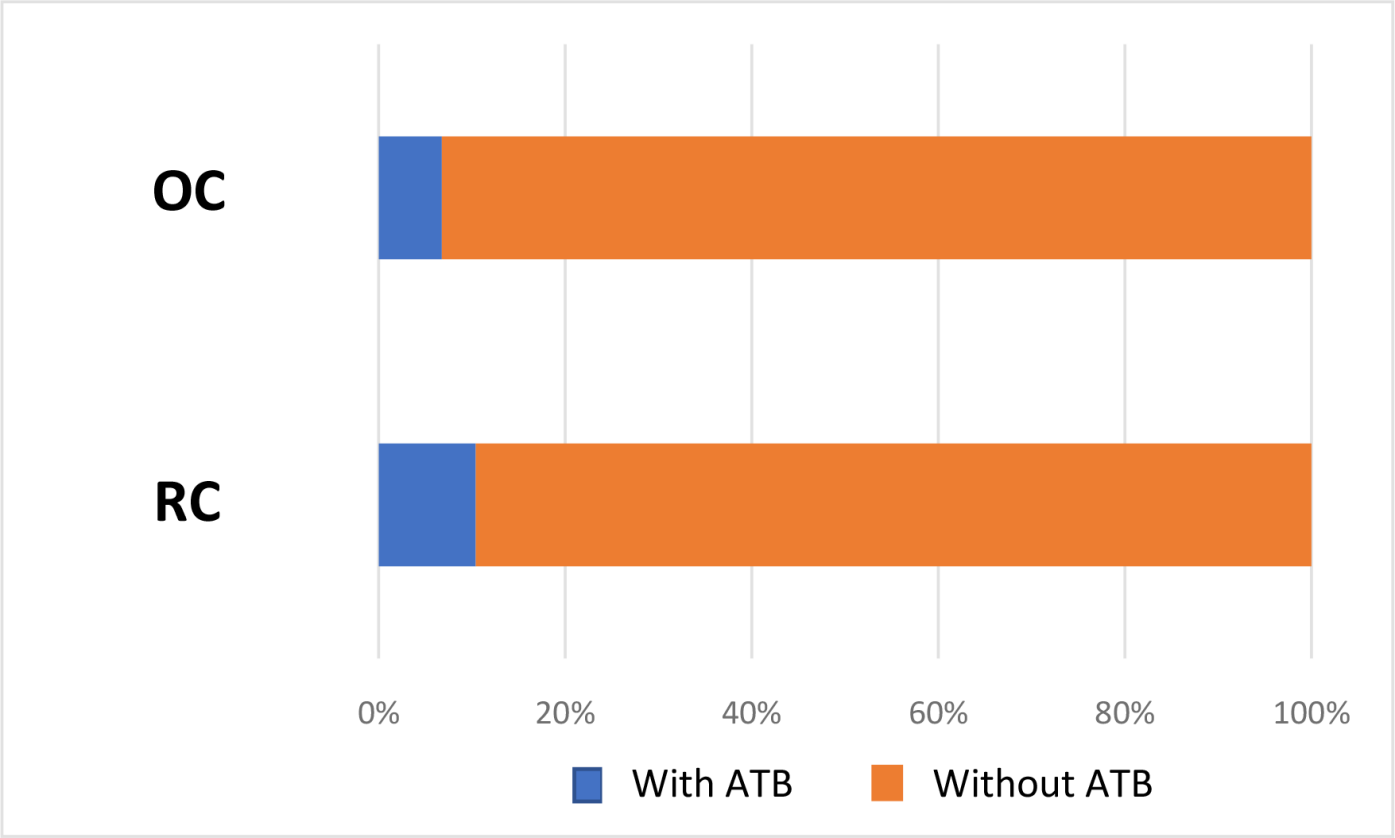
Rate of antibiotic prescription in GP consultations. ATB = antibiotic prescription. OC = office consultation. RC = remote consultation

Seven and half per cent of the 35 503 consultations included resulted in at least one ATB prescription, which represented 1946 OC and 704 RC (Figure 2). Of the 35 503 consultations selected for the main analysis, 10.41% of RCs resulted in at least one ATB prescription compared with 6.77% of OC in 2021 (odds ratio [OR] 1.60; 95% confidence interval [CI] = 1.46 to 1.75; *P*<0.01).

The multivariate analyses were carried out on the 2650 consultations resulting in at least one ATB prescription in 2021 (Table 2). Penicillin allergy was excluded from the multivariate analysis owing to many missing data (*n* = 801).

**Table 2.** Descriptive table of GP consultations with antibiotic prescription

Variable	*n* total (%)	*n* RC (%)	*n* OC (%)	Estimate	OR	95% CI	*P*
**Sex**	2650 (100)	704 (100)	1946 (100)				
Female (ref)	1839 (69.40)	516 (73.30)	1323 (67.99)				
Male	811 (30.60)	188 (26.70)	623 (32.01)	0.13	1.14	0.90 to 1.44	0.29
**Age, years**	2650 (100)	704 (100)	1946 (100)				
0–20	505 (19.06)	72 (10.23)	433 (22.25)	–0.92	0.40	0.29 to 0.54	<0.01
20–40 (ref)	836 (31.55)	330 (46.88)	506 (26.00)				
40–60	704 (26.57)	192 (27.27)	512 (26.31)	–0.56	0.57	0.45 to 0.73	<0.01
60–80	436 (16.45)	82 (11.65)	354 (18.19)	–1.04	0.35	0.25 to 0.49	<0.01
80–120	169 (6.38)	28 (3.98)	141 (7.25)	–1.31	0.27	0.16 to 0.44	<0.01
**LTC**	2650 (100)	704 (100)	1946 (100)				
Yes	536 (20.23)	116 (16.48)	420 (21.58)	–0.06	0.94	0.72 to 1.24	0.68
No (ref)	2114 (79.77)	588 (83.52)	1526 (78.42)				
**Reason for ATB**	2650 (100)	704 (100)	1946 (100)				
Angina	188 (7.09)	63 (8.95)	125 (6.42)	2.06	7.87	3.83 to 16.20	<0.01
Otitis media (ref)	257 (9.70)	10 (1.42)	247 (12.69)				
Sinusitis	274 (10.34)	73 (10.37)	201 (10.33)	1.76	5.83	2.85 to 11.98	<0.01
Other ENT	248 (9.36)	83 (11.79)	165 (8.48)	2.17	8.77	4.35 to 17.70	<0.01
Conjunctivitis	31 (1.17)	8 (1.14)	23 (1.18)	2.17	8.74	3.09 to 24.71	<0.01
Other eye infections	18 (0.68)	3 (0.43)	15 (0.77)	1.42	4.14	1.00 to 17.13	0.05
Dental diseases	52 (1.96)	12 (1.70)	40 (2.06)	1.67	5.34	2.07 to 13.72	<0.01
Pneumonias	73 (2.75)	4 (0.57)	69 (3.55)	0.31	1.36	0.41 to 4.55	0.62
Chronic bronchitis	41 (1.55)	5 (0.71)	36 (1.85)	1.41	4.09	1.29 to 13.06	0.02
Other respiratory infections	209 (7.89)	59 (8.38)	150 (7.71)	2.16	8.71	4.23 to 17.97	<0.01
Cystitis	486 (18.34)	211 (29.97)	275 (14.13)	2.88	17.85	8.94 to 35.63	<0.01
Prostatitis	66 (2.49)	21 (2.98)	45 (2.31)	2.30	9.87	4.14 to 23.48	<0.01
Pyelonephritis	86 (3.24)	27 (3.84)	59 (3.03)	2.28	9.76	4.37 to 21.76	<0.01
Genital infections	93 (3.51)	35 (4.97)	58 (2.98)	2.03	7.64	3.42 to 17.03	<0.01
Gut diseases	52 (1.96)	10 (1.42)	42 (2.16)	1.52	4.60	1.72 to 12.24	<0.01
Erysipelas	43 (1.62)	5 (0.71)	38 (1.95)	1.09	2.99	0.94 to 9.47	0.06
Other dermatological conditions	278 (10.49)	49 (6.96)	229 (11.77)	1.44	4.24	2.06 to 8.71	<0.01
Other	155 (5.85)	26 (3.69)	129 (6.63)	1.52	4.58	2.07 to 10.11	<0.01

ENT = ear, nose and throat. OC = office consultation. RC = remote consultation

More than two-thirds of the consultations with ATB prescription involved women. Women represented 73.29% of consultations with at least one ATB prescription in RC compared with 67.99% in OC. No correlation was demonstrated between sex and the mode of consultation for patients who had received a prescription for ATB.

Consultations of patients aged 0–20 years represented 19.06% of consultations with ATB prescription, 20–40 years 31.55%, 40–60 years 26.57%, 60–80 years 16.45%, and for people aged ≥80 years 6.38%. The RC rate for consultations with ATB prescription for age categories were 10.23%, 40.88%, 27.27%, 11.65%. and 3.98%, respectively. Consultations of 20–40 seconds with prescription of ATB were significantly more associated with RC than in other age categories (p < 0.01 compared to each age groupe).

About 20.23% of the consultations with ATB prescription involved patients with LTCs, of which 16.48% were carried out in RC, with no statistical correlation found between the use of RC in patients receiving an ATB and the presence of a LTC.

Diagnoses with ATB prescription are presented in Supplementary Table S2. Among consultations with ATB prescription, ENT infections were the most frequent cause of consultation, accounting for 36.49% of the total. Other main indications were urinary tract infection (24.08%), respiratory infections (12.19%), and dermatological infections (12.11%). In 5.85% of cases, the reason for prescribing an ATB was not identifiable in the patient record. Consultations with ATB prescription for pneumonitis and erysipelas were not correlated with RC. Other results of consultation with ATB prescription were significantly more correlated with RC than OC compared with the reference category. Consultation for urinary tract infection had the strongest correlation with RC than OC (p < 0.01 for respectively cystitis, prostatis and pyelonephritis) (Table 2).

## Discussion

### Summary

The rate of ATB prescription is significantly higher in RCs than in OCs in our sample of general practice consultations (p < 0.01). The 20–40 age group has benefited from the highest rate of ATB prescription in RCs. Concerning the results of consultations with ATB prescription, ENT and urinary infectious diseases were the most frequent consultation results, and the most important causes of ATB prescriptions. These results are important for the practice of general medicine as they lead to a reflection on the practice of teleconsultations and the risk of overprescription of ATBs. This study has contributed to filling the gap in the limited published data on the prescription habits of GPs in teleconsultation.

### Strengths and limitations

The main strengths of this study on medical practices among GPs lay in its originality, the data collection period of an entire year, and the size of the sample studied. To date, no study comparing the prescription of ATBs in general medicine in OCs with RCs has been carried out in France. The large sample size increased the power of this study. The ability to extract data using the Weda software resulted in only a small number of missing data.

Data collected were similar to those in the literature, with women receiving twice as many antibiotics as men (69% versus 31%) and RCs being more frequently used by patients aged <60 years.^2,7^ Only seven voluntary GPs were included owing to snowball effect and technical considerations. The recruited GPs' median age (43.8 years old) was under French median age of GPs (51.1 years old), and all of them were practising in urban areas. The RCs performed by participating doctors represented 19% of their total consultations, which is higher than the RC rate of French GPs in 2021. Studies have shown that younger, urban GPs make greater use of RC.^2,15^ This recruitment bias constituted the main limitation of the study.

The study period was marked by the COVID-19 pandemic, which affected GPs’ prescription behaviours. There was an increase in RC use associated with a drop in ATB prescriptions.^15,16^ It could be interesting to examine the evolution of ATB prescription trend in RCs compared with OCs over time.

### Comparison with existing literature

The rate of ATB prescription is significantly higher in RCs than in OCs in this study. This may be explained by the altered patient–physician communication in RC, the absence of a physical examination, and the impossibility of performing rapid diagnostic orientation tests in RCs.^17–19^ All these elements increase the risk of clinical uncertainty leading the practitioner to prescribe an ATB to reassure themselves or to respond to an explicit request from the patient.^20^

In other countries, studies comparing the prescription of ATBs in OCs with RCs have reported more mixed results. A Danish study in 2014 found greater ATB prescription in OC than in RC.^21^ However, the RCs analysed corresponded to telephone calls outside the opening hours of medical practices and the study population is among the lowest consumers of ATBs in Europe.^7^ Another study in the UK in 2002 found no difference in ATB prescription according to the mode of consultation.^17^ However, this study involved a small number of patients looking for a consultation appointment on the same day. Other studies corroborate our results with a higher prescription of ATBs in RCs than in OCs.^22,23^

The 20–40 age group has the highest rate of ATB prescription in RCs. This contrasts with a study by the French Public Health Agency in 2021 that found a higher rate of ATB prescription in older people in OCs.^7^ This difference is probably explained by a lower rate of teleconsultation in older people. Indeed, distributors of teleconsultation solutions have reported a greater use of RCs among patients aged <60 years.^24^ As this is an active population, access to the doctor via the RC proves to be a more practical solution.

At the extreme of the age range, <20 years and >60 years, consultations with ATB prescription are less frequent in RCs, which may be explained by the greater difficulty of access to RCs. It may also be owing to the importance given to the physical examination, and the close medical follow-up for these age groups.^25^ It would be interesting to know the number of transformations of RC into OC by the doctors themselves in these age groups.

ENT and urinary infectious diseases were the most frequent consultation results with ATB prescription. Consultation for urinary tract infection was the most correlated with RC. These results are in keeping with those already published.^26,27^ The anamnestic data on urinary tract infections are sensitive but not very specific, which seems to facilitate diagnosis even in the absence of physical examination. This facilitated diagnosis may lead to greater prescription of ATBs, even with the risk of numerous false positives in RC.

Our study found a higher proportion of ATB prescription in RC for many other consultation results probably because of clinical uncertainty. This increase in ATB prescriptions in RC, even for pathologies with probable viral aetiology, is in line with other studies.^20,28^ ATB prescription was less associated with RC for pneumopathies, erysipelas, and otitis media. Note that median age for erysipelas is >60 years and the prevalence peak of otitis media is <1 year, two age categories with lower use of RC. It is reasonable to assume that in these age categories the doctor attributes a greater role to the physical examination for otitis media and pneumonitis.^29–31^

### Implications for research and practice

This study has shown a higher rate of ATB prescription in RC than in OC. Consultation with ATB prescription for urinary infectious diseases, but also respiratory and ENT infectious diseases, which are often caused by viruses, are more associated with RC than OC despite the absence of physical examination. Patients between the ages of 20 and 40 years are the most affected by the phenomenon.

It would be interesting to analyse the prescription of ATBs according to consultation result, also including patients who have not been prescribed ATBs. Similarly, an analysis comparing ATB prescription in RC with OC, while considering the daily dose recommended by the World Health Organization (WHO), would also be relevant for a more comprehensive analysis of ATB overprescription in RC and the complications it may entail.

In response to certain abuses, French health policies have recently reduced the duration of sick leave prescriptions in RC to curtail associated health expenditure in 2023. To prevent further restriction on their prescribing habits, GPs should engage in a thorough reflection on their teleconsultation practices to mitigate the risks of antibiotic overprescription.

## Data are not available

The data used for this study belong to the patients and fall under the General Data Protection Regulation (GDPR)
